# HMGB1 Promotes Resistance to Doxorubicin in Human Hepatocellular Carcinoma Cells by Inducing Autophagy *via* the AMPK/mTOR Signaling Pathway

**DOI:** 10.3389/fonc.2021.739145

**Published:** 2021-10-27

**Authors:** Junhua Li, Wei Zhou, Qiang Mao, Dandan Gao, Lin Xiong, Xinyao Hu, Yongfa Zheng, Ximing Xu

**Affiliations:** ^1^Basic and Clinical Medical Research Center, Department of Gastroenterology, The First People’s Hospital of Jingmen, Jingmen, China; ^2^Department of Statistics, The First People’s Hospital of Jingmen, Jingmen, China; ^3^Department of Infectious Diseases, The First People’s Hospital of Jingmen, Jingmen, China; ^4^Department of Pathology, Renmin Hospital of Wuhan University, Wuhan, China; ^5^Cancer Center, Renmin Hospital of Wuhan University, Wuhan, China

**Keywords:** HMGB1, autophagy, hepatocellular carcinoma cell, drug resistance, doxorubicin, AMPK/mTOR pathway

## Abstract

Chemoresistance remains as a major hindrance in the treatment of hepatocellular carcinoma (HCC). High mobility group box protein 1 (HMGB1) enhances autophagic flux and protects tumor cells from apoptosis, which results in acquired drug resistance. However, the exact mechanisms underlying HMGB1-modulated autophagy in HCC chemoresistance remain to be defined. In the present study, we found that administration of doxorubicin (DOX) significantly promoted HMGB1 expression and induced HMGB1 cytoplasmic translocation in human HCC cell lines BEL7402 and SMMC7721, which enhanced autophagy that contributes to protecting HCC cells from apoptosis and increasing drug resistance. Moreover, we observed HMGB1 translocation and elevation of autophagy in DOX-resistant BEL7402 and SMMC7721 cells. Additionally, inhibition of HMGB1 and autophagy increased the sensitivities of BEL-7402 and SMMC-7721 cells to DOX and re-sensitized their DOX-resistant cells. Subsequently, we confirmed with HMGB1 regulated autophagy by activating the 5ʹ adenosine monophosphate-activated protein kinase (AMPK)/mTOR pathway. In summary, our results indicate that HMGB1 promotes acquired DOX resistance in DOX-treated BEL7402 and SMMC7721 cells by enhancing autophagy through the AMPK/mTOR signaling pathway. These findings provide the proof-of-concept that HMGB1 inhibitors might be an important targeted treatment strategy for HCC.

## Introduction

Liver cancer is considered to be the sixth most common cancer (1). The incidence and mortality of it rank the fourth and second among malignant tumors, respectively, which show a continuous upward trend in China (2, 3). Hepatocellular carcinoma (HCC) is the most common type of live cancer, accounting for approximately 75%–85% of cases (1, 4). Chemotherapy remains an indispensable comprehensive treatment for patients with postoperative or unresectable HCC at present (5, 6). Doxorubicin (DOX), a traditional chemotherapeutic agent for a wide variety of tumors, is a standard component for the treatment of advanced HCC. It demonstrates higher efficacy than other agents such as 5-fluorouracil, epirubicin, cisplatin, and etoposide (7, 8). However, the tendency to acquired resistance to DOX severely limits its clinical application in HCC therapy. DOX resistance involves multiple mechanisms, mainly related to drug accumulation (9), decreased DNA damage (10), and apoptosis signaling (11). Recently, the role of autophagy in DOX resistance has attracted a great deal of attention. Some studies have demonstrated that reversing DOX resistance *via* modulation of autophagy is a promising therapeutic strategy (12–14).

Autophagy is an essential cellular process that involves self-degradation of cellular proteins, damaged organelles, and lipid droplets *via* the lysosome, maintaining the energy balance and intracellular homeostasis (15). Recently, it was reported that autophagy is a significant contributor to chemoresistance in osteosarcoma cells and inhibition of autophagy enhances drug sensitivity of osteosarcoma cells (16). Autophagy has also been implicated in modulating sensitivity to oxaliplatin in human colorectal cancer cell lines (17). Some studies have shown that upregulation of autophagy promotes tumor cell survival and probably contributes to chemoresistance in liver cancer therapy (18, 19). These findings suggest that autophagy participates in the development of chemoresistance. However, little is still known about the underlying molecular mechanism of autophagy in regulating the development of chemotherapy resistance in HCC.

High mobility group box protein 1 (HMGB1), a well-known regulator of autophagy, is a highly conserved non-histone nuclear protein that has various biological functions in the nucleus such as DNA replication, recombination, transcription, and repair (20). In addition to its nuclear functions, HMGB1 in the cytoplasm acts as an extracellular signaling molecule that is closely associated with inflammation, cell proliferation and differentiation, and tumor progression (20, 21). Upregulation of HMGB1 expression has been unequivocally observed in various cancers such as HCC and lung cancer (22–24). Cytosolic translocation of HMGB1 and secretion of HMGB1 by tumor cells in response to chemotherapy are major factors in the disordered tumor microenvironment (25, 26). Many reports have demonstrated that subcellular localization and secretion of HMGB1 plays a major role as a positive regulator of autophagy in chemotherapy resistance in various cancers (26–29). However, the exact mechanism of HMGB1-mediated autophagy in the DOX resistance of HCC has not been clearly defined.

In this study, we investigated whether DOX augmented HMGB1 expression and induced HMGB1 translocation, whether the autophagy induced by DOX was regulated by HMGB1, and whether the changes in autophagy and HMGB1 protect HCC cells against DOX and facilitate the development of acquired DOX resistance. BEL7402 and SMMC7721 cells and DOX-resistant BEL7402 and SMMC7721 cells (BEL7402/DOX and SMMC7721/DOX cells, respectively) were used as the cell model. We found that HMGB1 expression and the associated autophagic flux were increased in response to DOX treatment in HCC cells, and autophagy modulated by HMGB1 protected HCC cells from DOX-induced apoptosis. Additionally, BEL7402/DOX and SMMC7721/DOX cells exhibited more autophagy and HMGB1 expression, and inhibition of autophagy and HMGB1 enhanced apoptosis sensitivity of DOX-resistant HCC cells to DOX. Moreover, we found that the 5ʹ adenosine monophosphate-activated protein kinase (AMPK)/mTOR signaling pathway was involved in these processes. Our data support HMGB1 as a potential molecular therapeutic target to enhance the efficacy of DOX in HCC.

## Materials and Methods

### Cell Culture and Establishment of Drug-Resistant Cell Lines

Human HCC cell lines BEL-7402 and SMMC-7721 were purchased from the Cell Bank of the Chinese Academy of Science (Shanghai, China). Cells were cultured in RPMI-1640 medium (Gibco, Invitrogen, CA, USA) supplemented with 10% fetal bovine serum (Thermo Fisher Scientific Inc., Shanghai, China) at 37°C in a humidified atmosphere with 5% CO_2_. DOX-resistant BEL-7402 and SMMC-7721 cell lines, BEL-7402/DOX and SMMC-7721/DOX, were established in our laboratory by selecting cells for resistance to increasing stepwise concentrations of DOX (Shanghai Shenggong Biological Engineering Co., Ltd., Shanghai, China) over 10 months until the cells survived in 1 µg/mL DOX as described previously (30). The half maximal inhibitory concentration (IC_50_) value was calculated with GraphPad Prism version 7.0 software, and the resistance index (RI) was calculated according to the following formulae: RI = (IC_50_ of drug-resistant cells)/(IC_50_ of parental cells), which was used as the relative indicator to evaluate drug resistance.

### Drug Sensitivity Measured by the MTT Assay

The sensitivity of HCC cells to DOX, expressed as the proliferation inhibition rate, was measured using the MTT assay. The cells were seeded at a density of 1 × 10^5^ cells per well in 96-well plates in 200 μl RPMI (Gibco, Invitrogen, CA, USA) and incubated at 37°C in a humidified atmosphere with 5% CO_2_ for 24 h. Then, the cells were treated with DOX at increasing concentrations of 0.1, 0.2, 0.4, 0.8, 1.6, and 3.2 µg/mL (five replicates for each concentration). After 48 h of culture at 37°C for adherence, the supernatants were removed and 20 µL per well MTT (Thermo Fisher Scientific, Shanghai, China) was added to the medium, followed by incubation for 2 h. Then, 150 µL per well of DMSO (Sigma-Aldrich, St. Louis, MO, USA) was added to dissolve the purple crystals. Subsequently, absorbance was determined at 490 nm and IC_50_ values were calculated by Graphpad Prism 7.0 software. The inhibition rate of cells was calculated by the following formula: (1-experimental blank absorbance value/control-blank absorbance value) × 100%. All experiments were repeated three times and the results are expressed as mean values.

### Construction of Vectors, siRNA, and Transfection Into Cells

BEL-7402 and SMMC7721 cells were seeded at 1 × 10^5^ cells per well in six-well plates and cultured to 80%–90% confluence. HMGB1-overexpressing vector pcDNA3.1-HMGB1 was obtained from Genechem Company (Shanghai, China) and transfected into HCC cells using Lipofectamine™ 2000 (Invitrogen, Carlsbad, CA, USA). Knockdown of HMGB1 was accomplished by specific small interfering RNA (siRNA). HMGB1-siRNA or negative control (NC) siRNA (GenePharma Company, Shanghai, China) were transfected into cell, using Lipofectamine™ 2000 in accordance with the manufacturers’ instructions. The siRNA sequences were as follows (31, 32): siHMGB1, sense strand 5ʹ-ccuguccauuggugauguutt-3ʹ and anti-sense strand 5ʹ-aacaucaccaauggacaggtt-3ʹ; siNC, sense strand 5ʹ-uucuccgaacgugucacgutt-3ʹ and anti-sense strand 5ʹ-acgugacacguucggagaatt-3ʹ. The working concentration of siRNA was 40 nmol/L. At 48 h after transfection, the culture medium was replaced and the cells were treated with the indicated concentrations of drugs for various periods.

### Western Blot Analysis

Cell lysates were prepared using RIPA lysis buffer containing 1 mM phenylmethylsulfonyl fluoride and 1% phosphatase inhibitor cocktail. The protein concentration was measured with a BCA assay (Beyotime Biotechnology, Shanghai, China). All samples were adjusted to equal protein content before analysis. Samples (20 µg total protein) from each group were separated by 12% sodium dodecyl sulfate-polyacrylamide gel electrophoresis and subsequently transferred onto PVDF membranes (Millipore, Billerica, MA, USA) under a constant current. Then, the membranes were blocked with 5% dry non-fat milk in TBST buffer for 2 hour at room temperature. The membrane was then incubated overnight at 4°C with a primary antibody diluted in TBST. The primary antibodies were as follows: polyclonal rabbit anti-human HMGB1, Beclin 1, LC3B, p62, AMPK, phosphorylated AMPK (p-AMPK), mTOR, phosphorylated mTOR (p-mTOR), and cleaved PARP (Affinity Biosciences, OH, USA), and antibodies against GAPDH (Cell Signaling Technology, Inc., MA, USA) and Lamin B (Boster Biological Technology Co. Ltd., Wuhan, China). After washing three times with TBST for 10 min each wash, the membrane was incubated with corresponding peroxidase-conjugated goat IgG (1:2,000 dilution, Boster Biological Technology Co. Ltd., Wuhan, China) as the secondary antibody for 1 h at room temperature. Enhanced chemiluminescent reagent (Applygen Technologies Inc., Beijing, China) was used for development. Protein bands were quantified and analyzed using the BandScan5.0 system. Each experiment was repeated three times and the results were averaged.

### Flow Cytometric Analysis of Apoptosis

Apoptosis was detected by annexin V-FITC and PI staining using an Apoptosis Detection kit (KeyGEN Bio TECH Co. Ltd., Nanjing, China) in accordance with the manufacturer’s instructions. Drug-treated cells were washed, collected, resuspended in PBS, and transferred to a flow cytometer tube after incubation with 500 μl Binding Buffer. Then, 5 µl annexin V-FITC and 5 µl PI were added, followed by incubation in the dark for 15 min at room temperature. Stained cells were analyzed by a CytoFLEX flow cytometer (Beckman Coulter, USA).

### Statistical Analysis

Statistical analyses were performed using SPSS22.0 software (IBM Corp., Armonk, NY, USA). Data are expressed as the mean ± standard deviation. Student’s t test was used for continuous variable comparison between two groups, and one-way ANOVA was adopted for multi-group comparison (Dunnett-*t* test or LSD-*t* test were used for multiple comparison). *P* < 0.05 was considered statistically significant.

## Results

### BEL7402/DOX and SMMC7721/DOX Cells Exhibit Stable Drug Resistance

**Figure 1:**
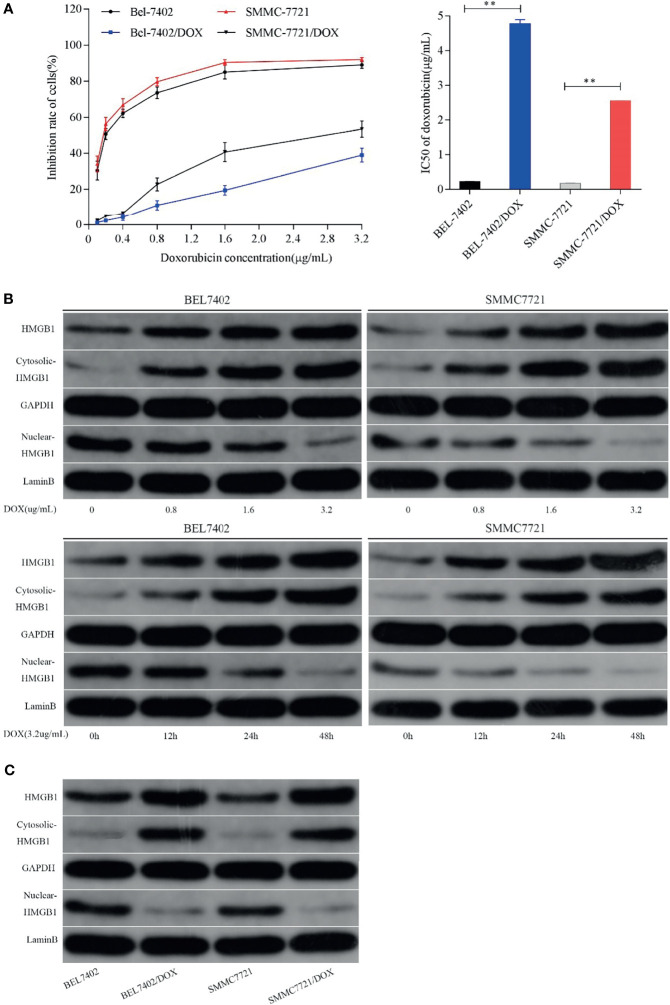
Chemotherapeutic treatment promotes HMGB1 expression and induces HMGB1 translocation. **(A)** Effect of DOX on the proliferation of BEL7402, BEL7402/DOX, SMMC7721, and SMMC7721/DOX cells, and DOX IC_50_ in these cells. The IC_50_ values of BEL7402/DOX and SMMC7721/DOX cells were higher than those of the parental cells, **P < 0.01. **(B)** Treatment with DOX increased the expression of HMGB1 and promoted HMGB1 translocation in a dose- and time-dependent manner. **(C)** Lysates of parental and DOX-resistant HCC cells were prepared to detect HMGB1. Total and cytoplasmic expression of HMGB1 were higher in DOX-resistant HCC cells.

To evaluate drug resistance of BEL7402/DOX and SMMC7721/DOX cells, the DOX IC_50_ of cells was determined by the MTT assay and RI indexes were calculated. The IC_50_ values of BEL7402 and BEL7402/DOX cells were 0.226 ± 0.004 and 4.776 ± 0.128 µg/mL, respectively, and those of SMMC7721 and SMMC7721/DOX cells were 0.175 ± 0.007 and 2.556 ± 0.002 µg/mL, respectively. As shown in **Figure 1A**, the RIs of BEL7402/DOX and SMMC7721/DOX cells were 21.1 and 14.6, respectively, suggesting that BEL7402/DOX and SMMC7721/DOX cells had exhibited stable DOX chemoresistance (33).

### DOX Treatment Promotes HMGB1 Expression and Induces HMGB1 Translocation in HCC Cell Lines

To determine whether HMGB1 expression was related to chemotherapy of HCC cells, we treated BEL-7402 and SMMC-7721 cells, which are commonly used in drug resistance experiments, with the chemotherapeutic drug DOX. DOX exerts anti-cancer effects by intercalating nucleotide bases, which depends on topoisomerase II enzyme, and inducing programmed cell death (34, 35). Here, BEL-7402 and SMMC-7721 cells were exposed to increasing concentrations of DOX or a fixed concentration (3.2 µg/mL, which determined by preliminary experiment) for 0, 12, 24, and 48 h. As shown in **Figure 1B**, western blot analysis revealed that DOX treatment led to a dose- and time-dependent increase in the total level of HMGB1 in both BEL-7402 and SMMC-7721 cells. Moreover, the cytosolic levels of HMGB1 were up-regulated, whereas HMGB1 expression in the nucleus was obviously reduced when the cells treated with DOX. Additionally, BEL-7402/DOX and SMMC-7721/DOX cells, the DOX-resistant sublines, showed relatively higher total and cytosolic levels of HMGB1 compared with parental cells (**Figure 1C**). These results suggested that DOX treatment induced expression and translocation of HMGB1 and that HMGB1 might be associated with drug resistance.

### Suppression of HMGB1 Increases the Sensitivity to DOX in HCC Cells

**Figure 2:**
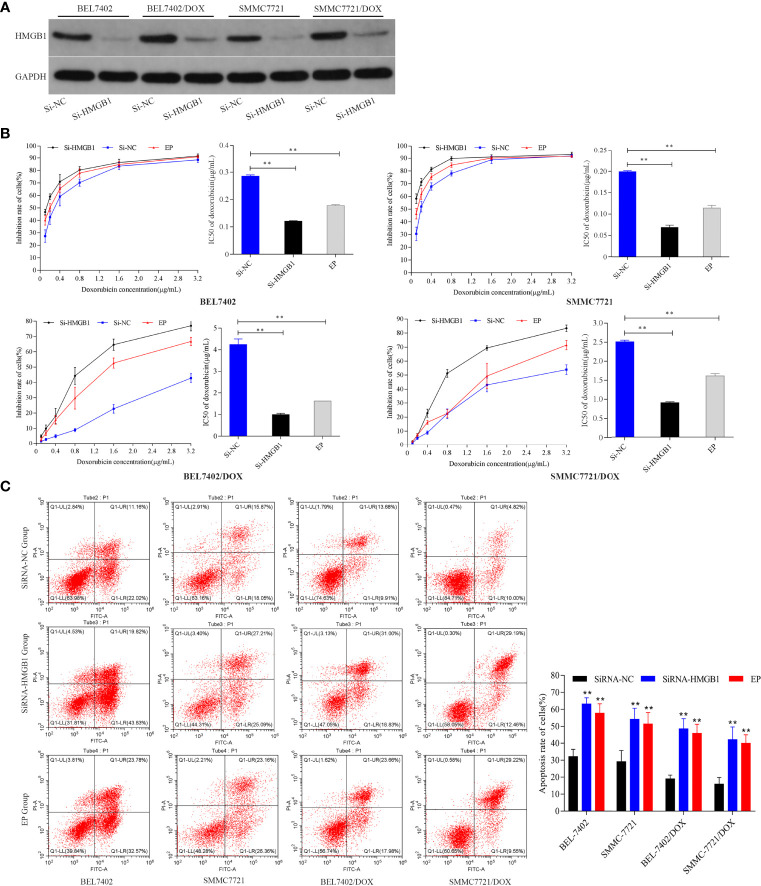
Inhibition of HMGB1 expression and cytoplasmic translocation increases sensitivity to DOX in HCC cells. **(A)** Compared with Si-NC cells, the level of HMGB1 was significantly decreased in Si-HMGB1 cells. **(B)** Suppression of HMGB1 expression and cytoplasmic translocation in HCC cells decreased the IC_50_ value of DOX. **P < 0.01 compared with Si-NC cells. **(C)** Inhibition of HMGB1 expression and cytoplasmic translocation enhanced apoptosis sensitivity of HCC cells to DOX. Apoptosis was analyzed by flow cytometric analysis of annexin-V/PI staining. **P < 0.01 compared with Si-NC cells.

To investigate the effect of HMGB1 induced by DOX on HCC cells, we analyzed the responses of BEL-7402 and SMMC-7721 cells and their DOX-resistant cells to DOX treatment after inhibition of HMGB1 expression and translocation. The cells were transfected with HMGB1 siRNA (Si-HMGB1) or negative control siRNA (Si-NC). HMGB1 expression was significantly lower in Si-HMGB1 cells than in Si-NC cells (**Figure 2A**). The cells were incubated with various concentrations DOX for 48 h after transfection with HMGB1 siRNA or pretreated with ethyl pyruvate (EP), a pharmacological inhibitor of HMGB1 cytoplasmic translocation (26). The IC_50_ values of cells transfected with HMGB1 siRNA or pretreated with EP were significantly lower than those of NC siRNA-transfected cells (**Figure 2B**), which indicated that the DOX sensitivities of BEL-7402 and SMMC-7721 cells were significantly increased by inhibition of HMGB1 expression and cytoplasmic translocation. Simultaneously, the apoptosis of cells, which were transfected with siRNA-HMGB1/siRNA-NC or pretreated with EP(10mM) 2h and then exposed to doxorubicin(3.2μg/mL) for 48h, was increased significantly after HMGB1 knockdown (**Figure 2C**), which suggested that suppression of HMGB1 enhanced apoptosis sensitivity in BEL-7402 and SMMC-7721 cells. Similar to the results in parental cells, transfection of BEL-7402/DOX and SMMC-7721/DOX cells with HMGB1 siRNA and pretreatment with EP rendered them largely more sensitive to DOX as indicated by decreases in IC_50_ values and increased apoptosis (**Figures 2B, C**).

### DOX Induces Autophagy That Protects HCC Cells From Apoptosis

**Figure 3:**
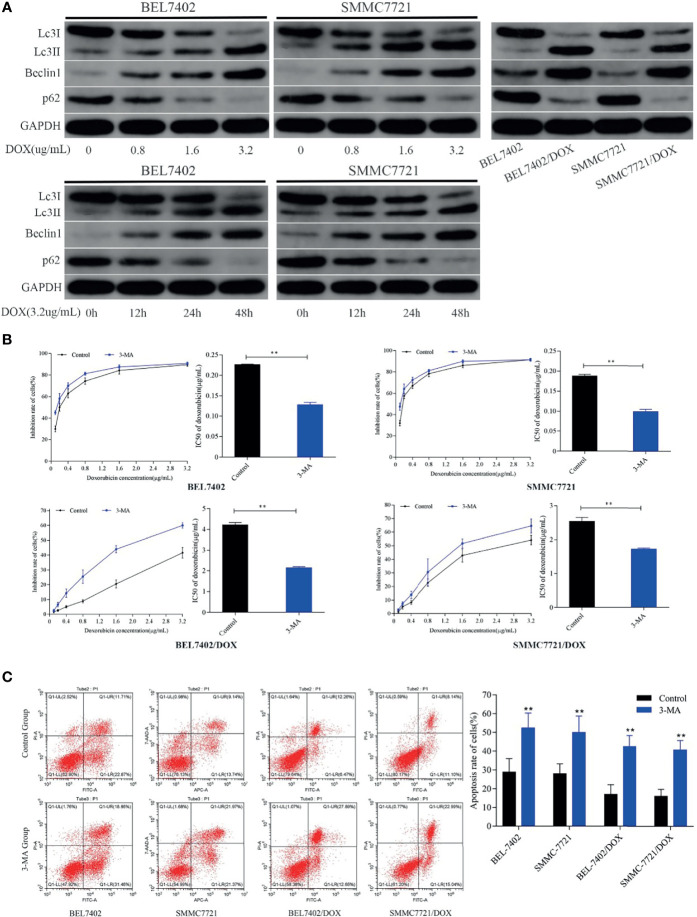
DOX induces autophagy that protects HCC cells from apoptosis and reduces the sensitivity of HCC cells to DOX. **(A)** Treatment with DOX promoted autophagy in a dose- and time-dependent manner in BEL7402 and SMMC7721 cells. Autophagy was also upregulated in DOX-resistant HCC cells. **(B)** Inhibition of autophagy promoted apoptosis and sensitivity of HCC cells to DOX. The DOX IC_50_ of parental and DOX-resistant HCC cells was significantly decreased by pretreatment with 3-MA. **(C)** Apoptosis rates of cells in the 3-MA group were remarkably higher than those of control cells both in parental and DOX-resistant HCC cells. **P < 0.01 compared with control cells.

To explore the effect of DOX on autophagy and the role of autophagy in chemotherapeutic drug resistance of HCC cells, we first detected the autophagy-related proteins Beclin 1, LC3-II, and p62, which are reliable markers of autophagy (36), in BEL-7402 and SMMC-7721 cells treated with various concentrations DOX for the indicated periods. As shown in **Figure 3A**, DOX markedly enhanced the levels of Beclin 1 and LC3-II, and reduced p62 expression in a time- and dose-dependent manner in BEL-7402 and SMMC-7721 cells.

These data indicated that treatment with chemotherapeutic drugs induced and increased autophagy in HCC cells.

Next, to determine whether DOX-induced autophagy was involved in drug resistance of HCC cells, we analyzed autophagy activity in two DOX-resistant cell lines: BEL-7402/DOX and SMMC-7721/DOX. Western blot analysis showed that the levels of LC3-II and Beclin 1 were higher and levels of p62 were lower in DOX-resistant cells than in parental cells (**Figure 3A**), which suggested that DOX-resistant HCC cells had an increased capacity for autophagy. Next, the parental and DOX-resistant HCC cells were pretreated with 3-MA (25μM) before incubating with doxorubicin(3.2μg/mL) for 48h. Cell proliferation inhibition rates were performed by MTT assay and IC50 was calculated. We found that the cytotoxicity and apoptosis were increased significantly when autophagy was inhibited by 3-MA in these kinds of cells as indicated by a decrease of IC_50_ and increase of apoptosis (**Figures 3B, C**). Furthermore, the sensitivity of BEL-7402/DOX and SMMC-7721/DOX cells to DOX was enhanced markedly, which indicated that the drug resistance of these cells was reversed by treatment with 3-MA (**Figure 3B**). Both parental and DOX-resistant cell lines showed potentiation of apoptosis after suppression of autophagy (**Figure 3C**).

These findings suggested that treatment with chemotherapeutic drug DOX induced autophagy in HCC cells, which protected HCC cells from DOX-induced apoptosis and contributed to the survival of HCC cells treated with DOX.

### HMGB1 Regulates DOX-Induced Autophagy in HCC Cells

**Figure 4:**
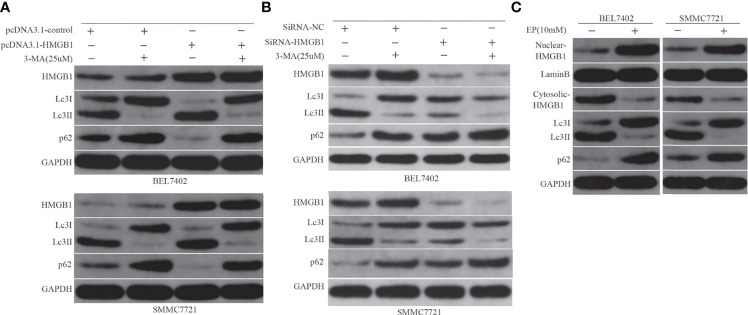
HMGB1 regulates autophagy in HCC cells. **(A)** Western blot analysis of HMGB1, LC3, and p62 levels in BEL7402 and SMMC7721 cells. **(B)** Western blot results showed inhibition of HMGB1 could significantly reduce autophagy in cells, which was more obvious when cells pretreated with 3-MA. **(C)** Nuclear and cytoplasmic HMGB1 and autophagy-related proteins were detected by western blotting.

Both HMGB1 and autophagy were induced by chemotherapy, which decreased sensitivity to the drug in HCC cells. Next, we investigated the relationship between them and examined whether HMGB1 is a direct regulator of autophagy. Previous studies have shown that starvation or other stresses facilitate translocation of HMGB1 to the cytoplasm and enhance autophagic flux (20, 37). In this study, BEL7402 and SMMC7721 cells were transfected with pcDNA3.1-control or pcDNA3.1-HMGB1 and then pretreated with or without 3-MA(25μM) for 12h before additional 48h incubated with doxorubicin(3.2μg/mL). HMGB1, Lc3 and P62 levels were assayed by western blot. We found that pcDNA3.1-HMGB1 vector significantly increased HMGB1 protein in the cells (**Figure 4A**). Moreover, western blot analysis showed that overexpression of HMGB1 increased the conversion levels of LC3-I to LC3-II and promoted the degradation of p62 compared with the pcDNA3.1-control group when cells were exposed to DOX. However, the LC3-II elevation and p62 degradation were abrogated by suppression of autophagy with 3-MA (**Figure 4A**). To further verify effect of HMGB1 on autophagy, the cells, BEL7402 and SMMC7721, were transfected with HMGB1 siRNA or negative control and then were treated with 3-MA and doxorubicin. And western blot results showed that inhibition of HMGB1 by transfecting with HMGB1 siRNA could significantly reduce autophagy in cells, which was more obvious when cells pretreated with 3-MA (**Figure 4B**). Furthermore, we observed changes of LC3-II and p62 expression in cells when cytosolic translocation of HMGB1 was inhibited by EP. BEL7402 and SMMC7721 cells were pretreated with or without EP(10mM) for 12h, and then incubated with doxorubicin (3.2μg/mL) for 48h. Nuclear and cytoplasmic HMGB1 and autophagy-related proteins were detected by western blotting. The results demonstrated that pretreatment with EP decreased the levels of LC3-II, but increased the level of p62 in cells before incubation with DOX (**Figure 4C**). Therefore, HMGB1 played an important role in the regulation of autophagy in HCC cells.

### HMGB1-Mediated Autophagy and Downregulated Apoptosis Induced by DOX Involve the AMPK/mTOR Pathway in HCC Cells

**Figure 5:**
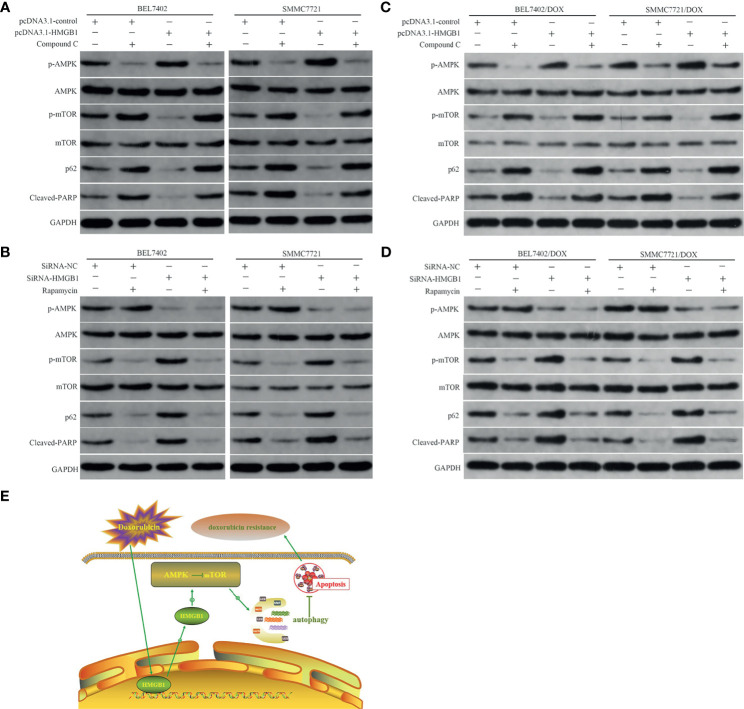
HMGB1-mediated autophagy that downregulates apoptosis in HCC cells involves the AMPK/mTOR pathway. **(A)** Western blot analysis of p-AMPK, p-mTOR, p62, and apoptosis-related protein cleaved PARP in BEL7402 and SMMC7721 cells transfected with pcDNA3.1-control or pcDNA3.1-HMGB1 and then pretreated with or without AMPK inhibitor Compound C (10μM). **(B)** Western blot analysis of p-AMPK, p-mTOR, p62, and cleaved PARP in BEL7402 and SMMC7721 cells transfected with siRNA-HMGB1 or siRNA-NC and then treated with or without mTOR inhibitor rapamycin(10 nM). **(C)** Western blot analysis of p-AMPK, p-mTOR, p62, and apoptosis-related protein cleaved PARP in DOX-resistant BEL7402 and SMMC7721 cells transfected with pcDNA3.1-control or pcDNA3.1-HMGB1 and then pretreated with or without AMPK inhibitor Compound C. **(D)** Western blot analysis of p-AMPK, p-mTOR, p62, and cleaved PARP in BEL7402/DOX and SMMC7721/DOX cells transfected with siRNA-HMGB1 or siRNA-NC and then treated with or without mTOR inhibitor rapamycin. **(E)** Model depicting the mechanism by which HMGB1 modulates doxorubicin resistance by inducing autophagy. Doxorubicin induces the cytosolic translocation of HMGB1, which promotes autophagy that decreases apoptosis and increases doxorubicin resistance by activating the AMPK/mTOR pathway.

AMPK is a highly conserved serine/threonine kinase that is widely distributed in eukaryotic cells, which is typically activated by a high AMP/ATP ratio to maintain energy homeostasis (38). Moreover, AMPK coordinates with many upstream and downstream molecules, such as LKBI, mTOR, 70 kDa ribosomal protein S6 kinase (p70S6K), Akt, and ULKI, and regulates apoptosis and autophagy (39–41). Mammalian rapamycin target protein mTOR—an atypical serine/threonine kinase—is an important downstream protein of AMPK and plays a “gating” role in regulation of autophagy by phosphorylation of p70S6K. Activated AMPK inactivates mTOR and the AMPK/mTOR pathway has been linked to actuation of autophagy (42–44). Thus, to determine whether the AMPK/mTOR pathway was involved in regulation of HMGB1-mediated autophagy in HCC cells, we measured AMPK and mTOR phosphorylation, markers of autophagy, p62, and apoptosis-related protein cleaved PARP in DOX-induced cells with or without AMPK inhibitor Compound C (10 μM) and mTOR inhibitor rapamycin (10 nM) treatments after transfection with the HMGB1 cDNA plasmid or HMGB1 siRNA. As shown in **Figure 5A**, compared with vector control cells, overexpression of HMGB1 significantly increased AMPK phosphorylation and obviously decreased the levels of p-mTOR, p62, and cleaved PARP in both BEL7402 and SMMC7721 cells. However, these effects were abolished by suppression of AMPK with Compound C. These results suggested that AMPK participated in promotion of autophagy by HMGB1, which downregulated apoptosis in HCC cells.

We also observed the role of mTOR in the abovementioned regulation process. Compared with the siRNA-NC group, depletion of HMGB1 by siRNA notably decreased p-AMPK and increased p-mTOR. Moreover, p62 degradation was weakened and cleaved PARP expression was enhanced in the siRNA-HMGB1 group, which further supported the role of AMPK in HMGB1-mediated autophagy and apoptosis of HCC cells. Additionally, when combined with mTOR inhibitor rapamycin in cells transfected with HMGB1 siRNA, the p-mTOR level was decreased obviously, p62 degradation was enhanced, and apoptosis was reduced significantly (**Figure 5B**). These results indicated that downregulation of p-AMPK after knockdown of HMGB1 did not promote p-mTOR expression, inhibit autophagy, or promote apoptosis when mTOR was blocked by rapamycin, which suggesting that mTOR was a downstream molecule of AMPK. Therefore, HMGB1 may regulate DOX-induced autophagy and reduce apoptosis through the AMPK/mTOR pathway in HCC cells. Similar to the results in parental cells, transfection of BEL7402/DOX and SMMC7721/DOX cells with HMGB1 cDNA plasmid improved activation of AMPK/mTOR pathway and autophagy (**Figure 5C**), and decreased the propensity for apoptosis. And the changes trend of these indexes were just opposite in HMGB1-knockdown DOX-resistant cells (**Figure 5D**). Together with data of the studies, we proposed a model in which doxorubicin induced the cytosolic translocation of HMGB1, which regulated autophagy that decreased apoptosis and increased drug resistance by activating the AMPK/mTOR pathway(**Figure 5E**).

## Discussion

In the present study, we demonstrated that DOX treatment markedly induced cytosolic HMGB1 translocation, HMGB1 expression, and autophagy in HCC cell lines. HMGB1-regulated autophagy contributed to the acquirement of DOX resistance by protecting HCC cells from apoptosis, and inhibition of HMGB1 or suppression of HMGB1 cytosolic translocation attenuated this autophagic protection in response to DOX. Additionally, we showed that activation of the AMPK/mTOR signaling pathway was involved in the process of HMGB1-mediated autophagy.

Acquired resistance is a major hindrance for the application of chemotherapeutic drugs to tumors. Numerous investigations have described that the mechanisms of DOX resistance include upregulation of multidrug resistance efflux pumps, topoisomerase, altered drug targets, and alterations in apoptosis signaling (45–48). However, the molecular mechanism of DOX resistance in HCC has not been fully elucidated. Currently, autophagy is considered as a novel clinical target to reverse DOX resistance. Studies have implied that autophagy is involved in several steps of HCC initiation and progression as well as therapeutic resistance (18, 19). Here, we found that BEL-7402 and SMMC-7721 cells underwent autophagy in a time- and dose-dependent manner when treated with DOX and drug-resistant sublines BEL-7402/DOX and SMMC-7721/DOX had an increased capacity for autophagy. Inhibition of autophagy by 3-MA potentiated the inhibitory effect of DOX on the proliferation of these cells, which was accompanied by significantly increased apoptosis. Additionally, autophagy inhibitor 3-MA partially reversed DOX resistance of BEL-7402/DOX and SMMC-7721/DOX cells by inhibiting autophagy. Our results suggested that DOX-induced autophagy protected HCC cells from apoptosis and was highly related to DOX resistance in these cells. Therefore, revealing the detailed mechanism of autophagy regulation may provide novel therapeutic options to improve chemotherapy efficacy.

HMGB1—a chromatin-associated nuclear protein—is a critical regulator of cell death and survival. Overexpression of HMGB1 is associated with the hallmarks of cancer, which included an unlimited replicative potential, angiogenesis, evasion of apoptosis, insensitivity to inhibitors of growth, inflammation, tissue invasion, and metastasis (20, 21). The activities of HMGB1 are related to its cellular localization. In the nucleus, HMGB1 binds to DNA and regulates nuclear events such as DNA replication, recombination, and repair. It is also actively secreted or passively released under various stimuli, such as injury, necrosis, hypoxia, and endotoxin, in different cell types (16, 20). Both endogenous and exogenous HMGB1 have been suggested to be important regulators of autophagy in tumor cells (49, 50). It was reported that anticancer agents doxorubicin induced HMGB1 upregulation in human osteosarcoma cells, and knockdown of HMGB1 restored the chemosensitivity of osteosarcoma cells *in vivo* and *in vitro* by inducing autophagy, an intracellular self-defense mechanism known to confer drug resistance (51). Pan et al. (26) found that HMGB1 is a crucial regulator of autophagy, which significantly contributes to docetaxel resistance in LAD cells. Wang et al. (50) showed that HMGB1 facilitates autophagic progression and reduces oxidative stress induced by DOX, which is a critical factor for the development of chemoresistance and tumorigenesis. This prompted us to investigate the relationship between HMGB1 and autophagy in chemotherapy resistance of HCC.

Here, we focused on the interaction between intracellular HMGB1 and autophagy in chemotherapy resistance to DOX. We observed that DOX treatment promoted HMGB1 expression and induced HMGB1 translocation in HCC cells and overexpression of HMGB1 by transfection with pcDNA3.1-HMGB1 increased the level of autophagy when HCC cells treated with DOX. However, this upregulation of autophagy was abolished by suppression of autophagy with 3-MA. Interestingly, we found that inhibition of autophagy by 3-MA was unable to increase the level of intracellular HMGB1, although autophagy is also regulated by the release of HMGB1 (52). Moreover, we observed that ethyl pyruvate (EP), an inhibitor of HMGB1 cytoplasmic translocation, attenuated DOX-induced autophagy. Our results showed that both HMGB1 upregulation and cytoplasmic translocation of HMGB1 enhanced the level of autophagy, which contributed to resistance against DOX when HCC cells were exposed to DOX. Furthermore, knockdown of HMGB1 or inhibition of HMGB1 cytoplasmic translocation increased sensitivity to DOX in BEL-7402 and SMMC-7721 cells and re-sensitized DOX-resistant BEL-7402/DOX and SMMC7721/DOX cells. Our findings obviously demonstrate that HMGB1 is a positive regulator of autophagy in HCC and mediates DOX resistance.

We further explored the molecular mechanism by which intracellular HMGB1 regulates autophagy. Previous studies have demonstrated the role of AMPK in viability, migration, invasiveness, and apoptosis of HCC cells (43, 53). Moreover, the AMPK/mTOR signaling pathway is involved in autophagy and AMPK negatively regulates mTOR and triggers autophagy flux. Thus, mTOR is suggested to be the predominate regulator of autophagy (42–44). Studies have showed that HMGB1 induced cardiomyocyte autophagy following acute myocardial infarction through activation of AMPK and inhibition of mTORC1 (54). Targeting autophagy enhances heat stress-induced apoptosis *via* the ATP-AMPK-mTOR axis in hepatocellular carcinoma (55). In the present study, we found that transfection with an HMGB1 cDNA plasmid promoted AMPK phosphorylation and reduced the level of mTOR phosphorylation, which were accompanied by increased autophagy and lower apoptosis. Suppression of AMPK with Compound C facilitated mTOR phosphorylation, inhibited autophagy, and enhanced apoptosis, even in HMGB1-overexpressing cells. These data suggested that AMPK participated in HMGB1-mediated promotion of autophagy, which downregulated apoptosis in HCC cells. Additionally, we observed that downregulation of p-AMPK by depletion of HMGB1 did not enhance p-mTOR expression, inhibit autophagy, or promote apoptosis of HCC cells when mTOR was blocked by rapamycin, which implied that mTOR was a downstream molecule of AMPK in the abovementioned regulation process. Taken together, these results suggest that HMGB1 regulates autophagy by activating the AMPK/mTOR pathway.

In summary, our study showed that both HMGB1 expression and cytoplasmic translocation of HMGB1 are enhanced by chemotherapy with DOX in HCC cell lines, which promotes autophagy that decreases apoptosis and increases drug resistance. HMGB1 facilitates autophagy by activating the AMPK/mTOR pathway. These results demonstrate that HMGB1 could be a potential target for HCC therapy. Further experiments are needed to clarify whether other downstream genes participate in the regulation process and to confirm our hypothesis in animal models *in vivo*.

## Data Availability Statement

The raw data supporting the conclusions of this article will be made available by the authors, without undue reservation.

## Author Contributions

All the authors have contributed greatly to this article. YZ and XX contributed to the conception of the study. JL and WZ performed the experiment and wrote the manuscript. DG and LX contributed significantly to analysis and manuscript preparation. QM performed the data analyses. XH helped perform the analysis with constructive discussions. All authors contributed to the article and approved the submitted version.

## Funding

This work was supported by the National Natural Science Foundation of China (no. 31971166 to XX) and Key Scientific Project of Jingmen (no. YFZD2017037 to JL).

## Conflict of Interest

The authors declare that the research was conducted in the absence of any commercial or financial relationships that could be construed as a potential conflict of interest.

## Publisher’s Note

All claims expressed in this article are solely those of the authors and do not necessarily represent those of their affiliated organizations, or those of the publisher, the editors and the reviewers. Any product that may be evaluated in this article, or claim that may be made by its manufacturer, is not guaranteed or endorsed by the publisher.

## References

[1] Bray F Ferlay J Soerjomataram I Siegel RL Torre LA Jemal A. Global Cancer Statistics 2018: GLOBOCAN Estimates of Incidence and Mortality Worldwide for 36 Cancers in 185 Countries. CA Cancer J Clin (2018) 68(6):394–424. doi: 10.3322/caac.21492 30207593

[2] Zheng RS Sun KX Zhang SW Zeng HM Zou XN Chen R. Report of Cancer Epidemiology in China, 2015. Zhonghua Zhong Liu Za Zhi (2019) 41(1):19–28. doi: 10.3760/cma.j.issn.0253-3766.2019.01.005 30678413

[3] Feng RM Zong YN Cao SM Xu RH. Current Cancer Situation in China: Good or Bad News From the 2018 Global Cancer Statistics? Cancer Commun (Lond) (2019) 39(1):22. doi: 10.1186/s40880-019-0368-6 31030667 PMC6487510

[4] Chen W Zheng R Baade PD Zhang S Zeng H Bray F. Cancer Statistics in China, 2015. CA Cancer J Clin (2016) 66(2):115–32. doi: 10.3322/caac.21338 26808342

[5] Chen S Cao Q Wen W Wang H. Targeted Therapy for Hepatocellular Carcinoma: Challenges and Opportunities. Cancer Lett (2019) 460:1–9. doi: 10.1016/j.canlet.2019.114428 31207320

[6] Galun D Srdic-Rajic T Bogdanovic A Loncar Z Zuvela M. Targeted Therapy and Personalized Medicine in Hepatocellular Carcinoma: Drug Resistance, Mechanisms, and Treatment Strategies. J Hepatocell Carcinoma (2017) 4:93–103. doi: 10.2147/JHC.S106529 28744453 PMC5513853

[7] Yeo W Mok TS Zee B Leung TW Lai PB Lau WY. A Randomized Phase III Study of Doxorubicin Versus Cisplatin/Interferon α-2b/Doxorubicin/Fluorouracil (PIAF) Combination Chemotherapy for Unresectable Hepatocellular Carcinoma. J Natl Cancer Inst (2005) 97(20):1532–8. doi: 10.1093/jnci/dji315 16234567

[8] Manov I Pollak Y Broneshter R Iancu TC. Inhibition of Doxorubicin-Induced Autophagy in Hepatocellular Carcinoma Hep3B Cells by Sorafenib—The Role of Extracellular Signal-Regulated Kinase Counteraction. FEBS J (2011) 278(18):3494–507. doi: 10.1111/j.1742-4658.2011.08271.x 21790999

[9] Lu YL Ma YB Feng C Zhu DL Liu J Chen L. Co-Delivery of Cyclopamine and Doxorubicin Mediated by Bovine Serum Albumin Nanoparticles Reverses Doxorubicin Resistance in Breast Cancer by Down-Regulating P-Glycoprotein Expression. J Cancer (2019) 10(10):2357–68. doi: 10.7150/jca.30323 PMC6584414 31258739

[10] Stefanski CD Keffler K McClintock S Milac L Prosperi JR. APC Loss Affects DNA Damage Repair Causing Doxorubicin Resistance in Breast Cancer Cells. Neoplasia (2019) 21(12):1143–50. doi: 10.1016/j.neo.2019.09.002 PMC6872841 31759252

[11] Xiang X Yuan D Liu Y Li J Wen Q Kong P. PIM1 Overexpression in T-Cell Lymphomas Protects Tumor Cells From Apoptosis and Confers Doxorubicin Resistance by Upregulating C-Myc Expression. Acta Biochim Biophys Sin (Shanghai) (2018) 50(8):800–6. doi: 10.1093/abbs/gmy076 30020405

[12] Chen C Lu L Yan S Yi H Yao H Wu D. Autophagy and Doxorubicin Resistance in Cancer. Anticancer Drugs (2018) 29(1):1–9. doi: 10.1097/CAD.0000000000000572 29099416

[13] Christowitz C Davis T Isaacs A van Niekerk G Hattingh S Engelbrecht AM. Mechanisms of Doxorubicin-Induced Drug Resistance and Drug Resistant Tumour Growth in a Murine Breast Tumour Model. BMC Cancer (2019) 19(1):757. doi: 10.1186/s12885-019-5939-z 31370818 PMC6670209

[14] Guo B Tam A Santi SA Parissenti AM. Role of Autophagy and Lysosomal Drug Sequestration in Acquired Resistance to Doxorubicin in MCF-7 Cells. BMC Cancer (2016) 16(1):762. doi: 10.1186/s12885-016-2790-3 27687594 PMC5043608

[15] Lohitesh K Chowdhury R Mukherjee S. Resistance a Major Hindrance to Chemotherapy in Hepatocellular Carcinoma: An Insight. Cancer Cell Int (2018) 18:44. doi: 10.1186/s12935-018-0538-7 29568237 PMC5859782

[16] Huang J Liu K Yu Y Xie M Kang R Vernon P. Targeting HMGB1-Mediated Autophagy as a Novel Therapeutic Strategy for Osteosarcoma. Autophagy (2012) 8(2):275–7. doi: 10.4161/auto.8.2.18940 PMC3336081 22301993

[17] Yang HZ Ma Y Zhou Y Xu LM Chen XJ Ding WB. Autophagy Contributes to the Enrichment and Survival of Colorectal Cancer Stem Cells Under Oxaliplatin Treatment. Cancer Lett (2015) 361(1):128–36. doi: 10.1016/j.canlet.2015.02.045 25749420

[18] Shi YH Ding ZB Zhou J Hui B Shi GM Ke AW. Targeting Autophagy Enhances Sorafenib Lethality for Hepatocellular Carcinoma *via* ER Stress-Related Apoptosis. Autophagy (2011) 7(10):1159–72. doi: 10.4161/auto.7.10.16818 21691147

[19] Sheng J Qin H Zhang K Li B Zhang X. Targeting Autophagy in Chemotherapy-Resistant of Hepatocellular Carcinoma. Am J Cancer Res (2018) 8(3):354–65. PMC5883089 29636994

[20] He SJ Cheng J Feng X Yu Y Tian L Huang Q. The Dual Role and Therapeutic Potential of High-Mobility Group Box 1 in Cancer. Oncotarget (2017) 8(38):64534–50. doi: 10.18632/oncotarget.17885 PMC5610024 28969092

[21] Tang D Kang R Zeh HJ 3rd Lotze MT. High-Mobility Group Box 1 and Cancer. Biochim Biophys Acta (2010) 1799(1-2):131–40. doi: 10.1016/j.bbagrm.2009.11.014 PMC2818552 20123075

[22] Ando K Sakoda M Ueno S Hiwatashi K Iino S Minami K. Clinical Implication of the Relationship Between High Mobility Group Box-1 and Tumor Differentiation in Hepatocellular Carcinoma. Anticancer Res (2018) 38(6):3411–8. doi: 10.21873/anticanres.12609 29848691

[23] Zhou J Yang Y Gan T Li Y Hu F Hao N. Lung Cancer Cells Release High Mobility Group Box 1 and Promote the Formation of Neutrophil Extracellular Traps. Oncol Lett (2019) 18(1):181–8. doi: 10.3892/ol.2019.10290 PMC6540031 31289487

[24] Huang CY Chiang SF Chen WT Ke TW Chen TW You YS. HMGB1 Promotes ERK-Mediated Mitochondrial Drp1 Phosphorylation for Chemoresistance Through RAGE in Colorectal Cancer. Cell Death Dis (2018) 9(10):1004. doi: 10.1038/s41419-018-1019-6 30258050 PMC6158296

[25] Zhan Z Li Q Wu P Ye Y Tseng HY Zhang L. Autophagy-Mediated HMGB1 Release Antagonizes Apoptosis of Gastric Cancer Cells Induced by Vincristine *via* Transcriptional Regulation of Mcl-1. Autophagy (2012) 8:109–21. doi: 10.4161/auto.8.1.18319 22108005

[26] Pan B Chen D Huang J Wang R Feng B Song H. HMGB1-Mediated Autophagy Promotes Docetaxel Resistance in Human Lung Adenocarcinoma. Mol Cancer (2014) 13:165. doi: 10.1186/1476-4598-13-165 24996221 PMC4125709

[27] Zhang R Li Y Wang Z Chen L Dong X Nie X. Interference With HMGB1 Increases the Sensitivity to Chemotherapy Drugs by Inhibiting HMGB1-Mediated Cell Autophagy and Inducing Cell Apoptosis. Tumour Biol (2015) 36(11):8585–92. doi: 10.1007/s13277-015-3617-6 26040768

[28] Chai W Ye F Zeng L Li Y Yang L. HMGB1-Mediated Autophagy Regulates Sodium/Iodide Symporter Protein Degradation in Thyroid Cancer Cells. J Exp Clin Cancer Res (2019) 38(1):325. doi: 10.1186/s13046-019-1328-3 31331356 PMC6647330

[29] Liu W Zhang Z Zhang Y Chen X Guo S Lei Y. HMGB1-Mediated Autophagy Modulates Sensitivity of Colorectal Cancer Cells to Oxaliplatin *via* MEK/ERK Signaling Pathway. Cancer Biol Ther (2015) 16(4):511–7. doi: 10.1080/15384047.2015.1017691 PMC4622507 25778491

[30] Chen S Wang Y Ruan W Wang X Pan C. Reversing Multidrug Resistance in Hepatocellular Carcinoma Cells by Inhibiting Extracellular Signal-Regulated Kinase/Mitogen-Activated Protein Kinase Signaling Pathway Activity. Oncol Lett (2014) 8(5):2333–9. doi: 10.3892/ol.2014.2521 PMC4186630 25295120

[31] Yue Y Zhou T Gao Y Zhang Z Li L Liu L. High Mobility Group Box 1/Toll-Like Receptor 4/Myeloid Differentiation Factor 88 Signaling Promotes Progression of Gastric Cancer. Tumour Biol (2017) 39(3):1010428317694312. doi: 10.1177/1010428317694312 28347236

[32] Cheng P Ma Y Gao Z Duan L. High Mobility Group Box 1 (HMGB1) Predicts Invasion and Poor Prognosis of Glioblastoma Multiforme *via* Activating AKT Signaling in an Autocrine Pathway. Med Sci Monit (2018) 24:8916–24. doi: 10.12659/MSM.912104 PMC6296343 30531692

[33] Snow K Judd W. Characterisation of Adriamycin- and Amsacrine-Resistant Human Leukaemic T Cell Lines. Br J Cancer (1991) 63(1):17–28. doi: 10.1038/bjc.1991.7 1989661 PMC1971666

[34] Kudo M. Systemic Therapy for Hepatocellular Carcinoma: 2017 Update. Oncology (2017) 93(Suppl 1):135–46. doi: 10.1159/000481244 29258077

[35] Usmani A Mishra A Arshad M Jafri A. Development and Evaluation of Doxorubicin Self Nanoemulsifying Drug Delivery System With Nigella Sativa Oil Against Human Hepatocellular Carcinoma. Artif Cells Nanomed Biotechnol (2019) 47(1):933–44. doi: 10.1080/21691401.2019.1581791 30888204

[36] Zhang B Yin X Sui S. Resveratrol Inhibited the Progression of Human Hepatocellular Carcinoma by Inducing Autophagy *via* Regulating P53 and the Phosphoinositide 3-Kinase/Protein Kinase B Pathway. Oncol Rep (2018) 40(5):2758–65. doi: 10.3892/or.2018.6648 30132535

[37] Tang D Kang R Livesey KM Cheh CW Farkas A Loughran P. Endogenous HMGB1 Regulates Autophagy. J Cell Biol (2010) 190(5):881–92. doi: 10.1083/jcb.200911078 PMC2935581 20819940

[38] Lin SC Hardie DG. AMPK: Sensing Glucose as Well as Cellular Energy Status. Cell Metab (2018) 27(2):299–313. doi: 10.1016/j.cmet.2017.10.009 29153408

[39] Liang P Jiang B Li Y Liu Z Zhang P Zhang M. Autophagy Promotes Angiogenesis *via* AMPK/Akt/mTOR Signaling During the Recovery of Heat-Denatured Endothelial Cells. Cell Death Dis (2018) 9(12):1152. doi: 10.1038/s41419-018-1194-5 30455420 PMC6242874

[40] Whang YM Kim MJ Cho MJ Yoon H Choi YW Kim TH. Rapamycin Enhances Growth Inhibition on Urothelial Carcinoma Cells Through LKB1 Deficiency-Mediated Mitochondrial Dysregulation. J Cell Physiol (2019) 234(8):13083–96. doi: 10.1002/jcp.27979 PMC13483273 30549029

[41] Liu J Long S Wang H Liu N Zhang C Zhang L. Blocking AMPK/ULK1-Dependent Autophagy Promoted Apoptosis and Suppressed Colon Cancer Growth. Cancer Cell Int (2019) 19:336. doi: 10.1186/s12935-019-1054-0 31871431 PMC6911288

[42] Hu Y Tao Y Hu J. Cannabinoid Receptor 2 Deletion Deteriorates Myocardial Infarction Through the Down-Regulation of AMPK-mTOR-P70s6k Signaling-Mediated Autophagy. Biosci Rep (2019) 39(4):BSR20180650. doi: 10.1042/BSR20180650 30923227 PMC6487266

[43] Gao L Lv G Li R Liu WT Zong C Ye F. Glycochenodeoxycholate Promotes Hepatocellular Carcinoma Invasion and Migration by AMPK/mTOR Dependent Autophagy Activation. Cancer Lett (2019) 454:215–23. doi: 10.1016/j.canlet.2019.04.009 30980867

[44] Suvorova II Pospelov VA. AMPK/Ulk1-Dependent Autophagy as a Key mTOR Regulator in the Context of Cell Pluripotency. Cell Death Dis (2019) 10:260. doi: 10.1038/s41419-019-1501-9 30886138 PMC6423002

[45] Cox J Weinman S. Mechanisms of Doxorubicin Resistance in Hepatocellular Carcinoma. Hepat Oncol (2016) 3(1):57–9. doi: 10.2217/hep.15.41 PMC4792121 26998221

[46] Nies AT König J Pfannschmidt M Klar E Hofmann WJ Keppler D. Expression of the Multidrug Resistance Proteins MRP2 and MRP3 in Human Hepatocellular Carcinoma. Int J Cancer (2001) 94(4):492–9. doi: 10.1002/ijc.1498 11745434

[47] Hyuga S Shiraishi M Hori A Hyuga M Hanawa T. Effects of Kampo Medicines on MDR-1-Mediated Multidrug Resistance in Human Hepatocellular Carcinoma HuH-7/PTX Cells. Biol Pharm Bull (2012) 35(10):1729–39. doi: 10.1248/bpb.b12-00371 23037162

[48] Ye CG Wu WK Yeung JH Li HT Li ZJ Wong CC. Indomethacin and SC236 Enhance the Cytotoxicity of Doxorubicin in Human Hepatocellular Carcinoma Cells via Inhibiting P-Glycoprotein and MRP1 Expression. Cancer Lett (2011) 304(2):90–6. doi: 10.1016/j.canlet.2011.01.025 21377266

[49] Su Z Wang T Zhu H Zhang P Han R Liu Y. HMGB1 Modulates Lewis Cell Autophagy and Promotes Cell Survival *via* RAGE-HMGB1-Erk1/2 Positive Feedback During Nutrient Depletion [Published Correction Appears in Immunobiology. 2018 Feb;223(2):258]. Immunobiology (2015) 220(5):539–44. doi: 10.1016/j.imbio.2014.12.009 25578401

[50] Wang L Zhang H Sun M Yin Z Qian J. High Mobility Group Box 1-Mediated Autophagy Promotes Neuroblastoma Cell Chemoresistance. Oncol Rep (2015) 34(6):2969–76. doi: 10.3892/or.2015.4278 26397184

[51] Huang J Ni J Liu K Yu Y Xie M Kang R. HMGB1 Promotes Drug Resistance in Osteosarcoma. Cancer Res (2012) 72(1):230–8. doi: 10.1158/0008-5472.CAN-11-2001 22102692

[52] Tang D Kang R Cheh CW Livesey KM Liang X Schapiro NE. HMGB1 Release and Redox Regulates Autophagy and Apoptosis in Cancer Cells. Oncogene (2010) 29:5299–310. doi: 10.1038/onc.2010.261 PMC2945431 20622903

[53] Sun Y Lei B Huang Q. SOX18 Affects Cell Viability, Migration, Invasiveness, and Apoptosis in Hepatocellular Carcinoma (HCC) Cells by Participating in Epithelial-To-Mesenchymal Transition (EMT) Progression and Adenosine Monophosphate Activated Protein Kinase (AMPK)/Mammalian Target of Rapamycin(mTOR). Med Sci Monit (2019) 25:6244–54. doi: 10.12659/MSM.915729 PMC6713035 31427562

[54] Foglio E Puddighinu G Germani A Russo MA Limana F. HMGB1 Inhibits Apoptosis Following MI and Induces Autophagy *via* Mtorc1 Inhibition. J Cell Physiol (2017) 232(5):1135–43. doi: 10.1002/jcp.25576 27580416

[55] Jiang J Chen S Li K Zhang C Tan Y Deng Q. Targeting Autophagy Enhances Heat Stress-Induced Apoptosis *via* the ATP-AMPK-mTOR Axis for Hepatocellular Carcinoma. Int J Hyperthermia (2019) 36(1):499–510. doi: 10.1080/02656736.2019.1600052 31007109

